# Enhancing the delivery and stability of lipid nanoparticle–dsRNA formulations in the RNAi-recalcitrant fall armyworm (*Spodoptera frugiperda*)

**DOI:** 10.3389/finsc.2026.1770055

**Published:** 2026-03-26

**Authors:** Marcel Kaarow, Leonie Graser, Eileen Knorr, Anton Windfelder, Pascal Geisler, Frank Steiniger, Markus Oberpaul, Andreas Vilcinskas, Christoph Hellmann

**Affiliations:** 1 Fraunhofer Institute for Molecular Biology and Applied Ecology (IME), Giessen, Germany; 2 Institute for Insect Biotechnology, Justus-Liebig University, Giessen, Germany; 3 Department of Diagnostic and Interventional Radiology (Experimental Radiology), University Hospital, Justus-Liebig University, Giessen, Germany; 4 Electron Microscopy Center, University Hospital, Friedrich Schiller University, Jena, Germany

**Keywords:** DsRNA uptake, FAW, lipid nanoparticles (LNPs), pest control, RNA interference (RNAi), *Spodoptera frugiperda*

## Abstract

The fall armyworm (FAW, *Spodoptera frugiperda*) is an invasive lepidopteran pest of staple crops. Its broad host range, ability to spread rapidly, and increasing resistance to pesticides pose a major threat to global food security. RNA interference (RNAi) offers a sustainable and targeted alternative to broad-spectrum chemical pesticides, but its efficacy is limited in lepidopterans primarily by the rapid degradation of double-stranded RNA (dsRNA) in the midgut and poor epithelial uptake. Here, we investigated lipid nanoparticles (LNPs) as a delivery strategy to enhance dsRNA stability and uptake in FAW larvae. LNP–dsRNA complexes (40–50 nm, +39 to +56 mV) were generated by the microfluidic mixing of a ternary lipid blend. Encapsulation protected dsRNA from degradation by gut enzyme extracts for up to 1 h, even under highly alkaline conditions (pH 11.5). The analysis of larvae exposed to Cy3-labeled dsRNA by fluorescence microscopy demonstrated that LNPs improved internal distribution beyond the gut lumen, whereas unformulated dsRNA mainly accumulated at the peritrophic membrane. These results indicate that LNPs resist the gut environment and overcome limited systemic uptake, the two major physiological barriers to RNAi in lepidopterans, enabling the more efficient delivery of dsRNA. This study establishes a lipid nanoparticle-based dsRNA delivery platform that overcomes key physiological barriers in FAW, providing a prerequisite for future *in vivo* gene knockdown and efficacy studies.

## 1 Introduction

Agricultural production must meet rising global food demands despite the declining availability of arable land and increasing pressures from climate change, soil degradation, and biodiversity loss (1, 2). Insect pests intensify these challenges by causing substantial crop losses and transmitting plant pathogens (3, 4). Farmers currently rely on broad-spectrum chemical pesticides or bioinsecticides such as *Bacillus thuringiensis* to prevent infestations (5–7) but these can harm beneficial species and other non-target organisms, and persist in the environment (8–10). Global pesticide use therefore continues to increase despite regulatory efforts to restrict hazardous chemicals (11), creating a demand for selective, environmentally friendly, and publicly acceptable pest-control strategies.

RNA-based plant protection is a promising strategy targeting specific pests with agents that break down rapidly and safely in the environment (12–14). RNA interference (RNAi), a conserved gene-silencing mechanism in which double-stranded RNA (dsRNA) is processed into small interfering RNAs (siRNAs) that target complementary mRNA (15, 16), has been used to suppress essential genes in multiple insect pests (16–21). Current delivery strategies rely on host-induced gene silencing (HIGS), where the dsRNA is expressed in genetically modified plants, or spray-induced gene silencing (SIGS), where it is applied to the plant surface (22). The first HIGS-based commercial product was Monsanto’s MON 87411 maize, which expresses dsRNA targeting the vesicular trafficking component Snf7 in the western corn rootworm *Diabrotica virgiferavirgifera* (23, 24). This crop is approved in the USA, but the broader deployment of HIGS remains constrained by GMO regulations (25–27). The first SIGS-based commercial product was ledprona (marketed as Calantha), approved by the US Environmental Protection Agency as a sprayable dsRNA-based insecticide (28) targeting the proteasome subunit PSMB5 in the Colorado potato beetle *Leptinotarsa decemlineata* (29). However, RNAi efficacy is highly variable across insect taxa, with lepidopterans in particular showing poor responses mainly due to their strongly alkaline midgut environment and abundant RNases that rapidly degrade unprotected dsRNA (30–35).

Despite its potential as a species-restricted control approach, RNAi is inefficient against the fall armyworm (FAW; *Spodoptera frugiperda* J.E. Smith; Lepidoptera: *Noctuidae*), a polyphagous pest infesting more than 350 plant species, including maize, rice, cotton, and sorghum (36, 37). FAW is native to the Americas, where it causes maize yield losses of 17.7 million tons/year (38), but it has spread to more than 80 countries across Africa, Asia, Europe and the Pacific through long-distance migration and human-mediated dispersal (38–40). It causes maize losses valued at US$2.5–6.2 billion/year in Africa alone (38). Climate projections indicate that FAW will continue expanding into temperate regions (38), increasing the need for innovative and sustainable control methods.

Formulations that enhance or bypass conventional dsRNA entry pathways, including SID-1-like channels, clathrin-mediated endocytosis, and receptor-mediated uptake, provide one avenue to address the limitations of RNAi, especially in targets such as FAW where SID-1-like transporters are scarce or absent (41). For example, encapsulated RNA molecules could be designed to fuse with cellular membranes or exploit alternative internalization mechanisms, thus increasing the efficiency of intracellular delivery in recalcitrant species. Lipid nanoparticles (LNPs) are widely used in nucleic acid therapeutics, including the first two FDA-approved SARS-CoV-2 vaccines (Moderna mRNA-1273 and Pfizer BNT162b2), where they enable safe, efficient mRNA delivery (42–45). These same physicochemical principles could be exploited for the delivery of dsRNA to agricultural pests. LNPs protect dsRNA from rapid enzymatic degradation, enhance cellular uptake, and improve stability under harsh physiological conditions (46).

Here, we describe the formulation of LNPs using scalable, technical-grade lipids (47). The microfluidic mixing of a cationic lipid (DDAB) for dsRNA complexation, a PEGylated lipid for colloidal stability, and a natural phospholipid for structural integrity ensures reproducible nanoparticle characteristics. We investigated the ability of these cost-effective LNP formulations to enhance the RNAi-based control of FAW by evaluating their physicochemical properties and resistance to FAW gut enzyme extracts. Using Cy3-labeled dsRNA, we also assessed whether LNP encapsulation improves gut barrier translocation and systemic distribution in larvae. This allowed us to validate LNPs as protective and functional carriers that overcome key physiological barriers affecting the efficacy of RNAi in lepidopteran pests.

## 2 Materials and methods

### 2.1 Rearing of *Spodoptera frugiperda*

A FAW laboratory colony was maintained in a climate-controlled chamber at 26 ± 1 °C and 60–65% relative humidity, with a 16-h photoperiod. Egg masses were placed on an artificial diet in vented Petri dishes until hatching. Newly emerged larvae (L1) were individually transferred to plastic cups containing 7 mL artificial diet. Larvae were reared until pupation (11–14 days), after which pupae were sexed and kept in separate cups until adult emergence. Adults were held in plastic containers lined with paper substrate and supplied with honey-water via cotton wicks. Oviposition occurred after 3–4 days and egg masses were collected to maintain the colony.

Larvae were reared on a pinto bean-based artificial diet adapted from Nalin (48), comprising a vitamin-antibiotic solution and a dry diet mixture. The vitamin-antibiotic solution was prepared by dissolving 10 g Vanderzant vitamin mixture for insects and 0.25 g tetracycline (both Merck) in 50 mL deionized water (ddH_2_O). The dry diet mixture (400 g) consisted of finely ground pinto beans, wheat seedlings, soybeans, casein, torula yeast, ascorbic acid, methyl-4-hydroxybenzoate, and sorbic acid, combined at a ratio of 125:100:50:50:62.5:6:5:3 dry weight. This dry mix was thoroughly blended with the vitamin-tetracycline solution and 850 mL of ddH_2_O to create a homogeneous base for insect rearing. Separately, 35 g of agar (Agar-Agar Kobe I, Carl Roth) was dissolved in 1100 mL ddH_2_O and heated to boiling point in a microwave. After cooling to ~70 °C, the liquid agar was incorporated into the blended diet base, and the entire mixture was homogenized using a blender and poured into trays and cups to solidify. Diet portions were allowed to dry under sterile airflow, irradiated with UV light to reduce surface contamination, sealed, and stored at 4 °C.

For feeding experiments, diet pellets (~6 × 2.5 mm) were prepared by dispensing 75-µL aliquots of the liquid diet into a 96-well plate covered with breathable film, followed by lyophilization. These pellets provided standardized diet units for L1 larvae in the uptake and delivery assays.

### 2.2 Enzyme extract preparation

Midgut lumen extracts were prepared from 15 FAW larvae (L5), which were chilled on ice for 20 min and dissected under sterile conditions. The head and terminal abdominal segments were removed, and the midgut was exposed by opening the cuticle. Foregut and hindgut tissues were excised, and luminal contents were collected into 3 mL ice-cold phosphate-buffered saline (PBS). As a diet control, 270 mg freshly prepared artificial diet was resuspended in 2 mL PBS and processed in parallel. Samples were vortexed and centrifuged (8000 × g, 10 min, 4 °C) to remove particulate material. Supernatants were passed through 0.2-µm PTFE syringe filters and stored at –80 °C.

To compare the activity of commercial RNase III (ShortCut RNase III, NEB) and FAW midgut lumen extracts, all reactions were standardized based on total protein concentration. Protein levels were quantified using the Pierce BCA Protein Assay Kit (Thermo Fisher Scientific). Bovine serum albumin (BSA) standards were prepared in PBS for midgut and diet extracts, and in distilled water for RNase III samples to match the corresponding buffer environments. Absorbance was measured at 562 nm using a multimode plate reader (Cytation 5, Agilent). Blank-corrected values were fitted using a fourth-degree polynomial standard curve, as recommended by the BCA kit protocol, and sample protein concentrations were interpolated accordingly. Final concentrations were adjusted for dilution factors and reported as the mean of replicate measurements.

### 2.3 LNP–dsRNA formulation

LNPs were prepared by microfluidic mixing of an aqueous solution of 500-bp dsRNA (Genolution) and a ternary lipid blend dissolved in ethanol (47). Formulations were produced using two syringe pumps and a 3D serpentine microfluidic mixing chip (Fluidic 1079, microfluidic ChipShop). The aqueous phase contained dsRNA diluted in nuclease-free water (pH 6–7), whereas the organic phase consisted of (di-*n*-dodecyl)dimethylammonium bromide (DDAB; Thermo Fisher Scientific), PEG-lipid Kolliphor HS 15 (Sigma-Aldrich), and egg lecithin (Carl Roth) mixed at a 2:1:1 molar ratio. Each lipid was prepared as a 100 mM stock solution in ethanol. Because the formulation contained the constitutively cationic lipid DDAB, complex formation did not require acidic buffer conditions.

The components were mixed at a total flow rate of 12 mL/min with a 9:1 aqueous-to-organic flow rate ratio (FR_aq_ = 10.8 mL/min; FR_org_ = 1.2 mL/min). The final dsRNA concentration was 0.2 g/L, corresponding to ~0.615 µM dsRNA. Lipid quantities were calculated based on a lipid/phosphate (L/P) ratio of 4, and the required volumes of each lipid stock were combined with ethanol to obtain the 12-mL organic phase used during the 10-min mixing period. We did not include dialysis or buffer exchange steps during or after microfluidic formulation. The resulting dispersions were used directly for subsequent characterization and experiments.

Both phases were filter-sterilized (aqueous, 1.0/0.45-µm GF/PET; organic, 0.2-µm PTFE) and equilibrated at 60 °C for 45 min prior to formulation. The filtered solutions were loaded into syringes and introduced into the mixing chip under continuous flow. The initial 1–2 mL of output was discarded to eliminate dead volume, and subsequent fractions were pooled and stored at 5 °C.

### 2.4 LNP characterization

Freshly prepared LNP formulations (0.2 mg/mL dsRNA) were equilibrated to room temperature. Particle size and size distribution were measured by multi-angle dynamic light scattering (MADLS) using a Zetasizer Ultra with DTS0012 disposable cells (both from Malvern Panalytical) at 25 °C under standard dispersant settings (RI 1.33, viscosity 0.887 cP). For Cy3-labeled formulations, samples were collected after fluorescence microscopy, stored at 5 °C, and diluted to 16 µg/mL dsRNA before measurement. An unlabeled LNP control of identical concentration and storage history was analyzed in parallel.

Zeta potential was determined on the same instrument using a DTS1070 folded capillary cell (Malvern Panalytical) in combined size-zeta mode. Size distributions were recorded immediately before and after zeta measurements (five runs per sample) to confirm particle stability.

For cryogenic transmission electron microscopy (cryo-TEM), we applied 7 µL samples to both sides of gold grids covered with perforated gold film (UltrAuFoil 1.2/1.3; Quantifoil Micro Tools). Excess sample was automatically blotted from the back of the grid with strips of filter paper for 1 s. The samples were rapidly plunge-frozen in liquid ethane (cooled to –180 °C) in a Cryobox (Carl Zeiss). Excess ethane was removed with filter paper. Samples were transferred immediately to a Gatan 626 cryo-transfer holder and into the pre-cooled cryo-electron microscope (Philips CM 120) operated at 120 kV. Images were viewed under low-electron-dose conditions and recorded using an F216 2K complementary metal-oxide semiconductor (CMOC) camera (TVIPS). Four images were averaged to reduce noise.

### 2.5 Stability assay

The integrity of dsRNA was assessed by 2% (w/v) agarose gel electrophoresis in TAE buffer at 80 V for 45 min. Samples were mixed with SYBR Gold stain and 10× loading buffer without sodium dodecylsulfate (SDS). We used 100-bp DNA ladder (NEB) as a molecular weight reference. Gels were imaged on a Gel Doc XR system (Bio-Rad Laboratories), and band intensities were evaluated to compare the degradation of naked and LNP-formulated dsRNA.

Enzymatic degradation of naked and LNP-formulated dsRNA was evaluated in 60-µL reactions consisting of 40 µL dsRNA (0.2 g/L) and 10 µL of enzyme solution (either FAW midgut lumen extract in PBS or commercial RNase III with supplied cofactors in nuclease-free water) standardized to 1 mg/mL total protein. Nuclease-free water was added to reach the final volume, and water-only controls were prepared by replacing the enzyme solution. The reaction pH was adjusted to the target conditions and verified using indicator strips. All samples were incubated at 32 °C to ensure consistent and robust RNase activity.

For conditions requiring LNP disruption, reactions were split after incubation and one half was treated with SDS before electrophoresis. Prior to loading, samples were mixed with SDS-free loading buffer and SYBR Gold stain. Treated and untreated aliquots were analyzed by agarose gel electrophoresis as described above to compare dsRNA protection and release.

### 2.6 Delivery of LNPs containing Cy3-labeled dsRNA

We used a separately prepared LNP batch containing Cy3-labeled dsRNA for feeding and fluorescence imaging experiments because fluorescence labeling necessitated independent formulation. The particle size distribution of this batch is shown in **Supplementary Figure 1**. Cy3-labeled dsRNA (500 bp, non-targeting; Genolution) was prepared using the LabelIT siRNA Tracker Kit (Mirus Bio) and purified by ethanol precipitation. Labeling efficiency and final concentration were verified by spectrophotometry. For LNP delivery, the labeled dsRNA was encapsulated using the microfluidic mixing protocol as above.

Two complementary delivery approaches were tested: an *in vivo* feeding assay and an *ex vivo* midgut assay. For the feeding assay, lyophilized artificial diet pellets were soaked in 5 µL dsRNA-Cy3 or LNP–dsRNA-Cy3 (0.1 g/L) made up to 50 µL with water. Starved L1 larvae (n = 5 per treatment) were allowed to feed for 4 h. Diet consumption was not quantified on a per-larva basis because the assay was designed for the qualitative assessment of uptake and tissue localization rather than ingestion efficiency. To minimize variability, larvae were starved prior to feeding, exposed to identical diet volumes, and monitored for feeding behavior during the assay. Larvae were then washed and frozen. Prior to cryo-sectioning, larvae were screened under low-magnification fluorescence to confirm ingestion, and individuals showing detectable gut fluorescence were selected for downstream imaging. This criterion was applied uniformly across treatments. Frozen larvae were embedded in OCT and cryo-sectioned at 16 µm. Sections were mounted with Fluoroshield containing DAPI (Thermo Fisher Scientific). For the *ex vivo* assay, midguts from L4 larvae (n = 4) were opened sagittally, rinsed with PBS, and spotted with 4 µL dsRNA-Cy3 or LNP–dsRNA-Cy3 (0.1 µg/µL). Tissues were incubated for 1 h at 32 °C in humidified chambers, embedded in OCT, and cryo-sectioned as above.

### 2.7 Fluorescence microscopy and quantification

Sections were imaged using a DM5000B microscope (Leica Microsystems). Identical exposure settings were applied across treatments. Bright-field images were captured using 4×, 10× and 20× objectives with fixed aperture (2) and gain (1), applying exposure times of 10 ms (4× and 10×) or 63 ms (20×) and transmission light field settings of 33 or 12, respectively. Cy3 fluorescence was acquired using the N3 filter set (Ex/Em 546/600 nm) with constant gain (5), incident light field (6), and full-field illumination (FIM 100%). Two Cy3 exposure regimes were used: a low-exposure setting (1 s) for quantitative analysis and a high-exposure setting (5 s) for enhanced signal visualization. DAPI fluorescence was recorded using the A4 filter set (Ex/Em 365/470 nm) with 250 ms exposure, gain 1, incident light field 6, and FIM 100%. Bright-field images were inverted before channel merging to improve structural contrast in the composite. For quantitative analysis, fluorescence intensity was measured from raw, fixed-exposure grayscale images in FIJI/ImageJ. Mean grey values (0–4095) were extracted from manually defined regions of interest (ROIs) placed within the hemocoel on the basis of DAPI staining, with background subtraction from an external ROI. Intensity data were exported for downstream statistical analysis.

### 2.8 Statistical analysis

Fluorescence intensity data (mean gray values from raw images acquired at fixed exposure settings) were quantified in FIJI/ImageJ. Because the experiment was designed as an imaging-based uptake assessment with matched treatment comparisons, statistical analysis was applied using paired t-tests in Microsoft Excel. A significance threshold of α = 0.05 was used (*p < 0.05; **p < 0.01; n.s., not significant). The sample size (n = 5 per treatment) reflects the technically demanding nature of cryo-sectioning and microscopy workflows and is consistent with exploratory imaging studies intended to demonstrate qualitative differences in tissue distribution rather than population-level feeding variability.

## 3 Results

### 3.1 Alkaline pH enhances FAW midgut RNase activity, leading to accelerated dsRNA degradation compared to commercial RNase III

**Figure 1:**
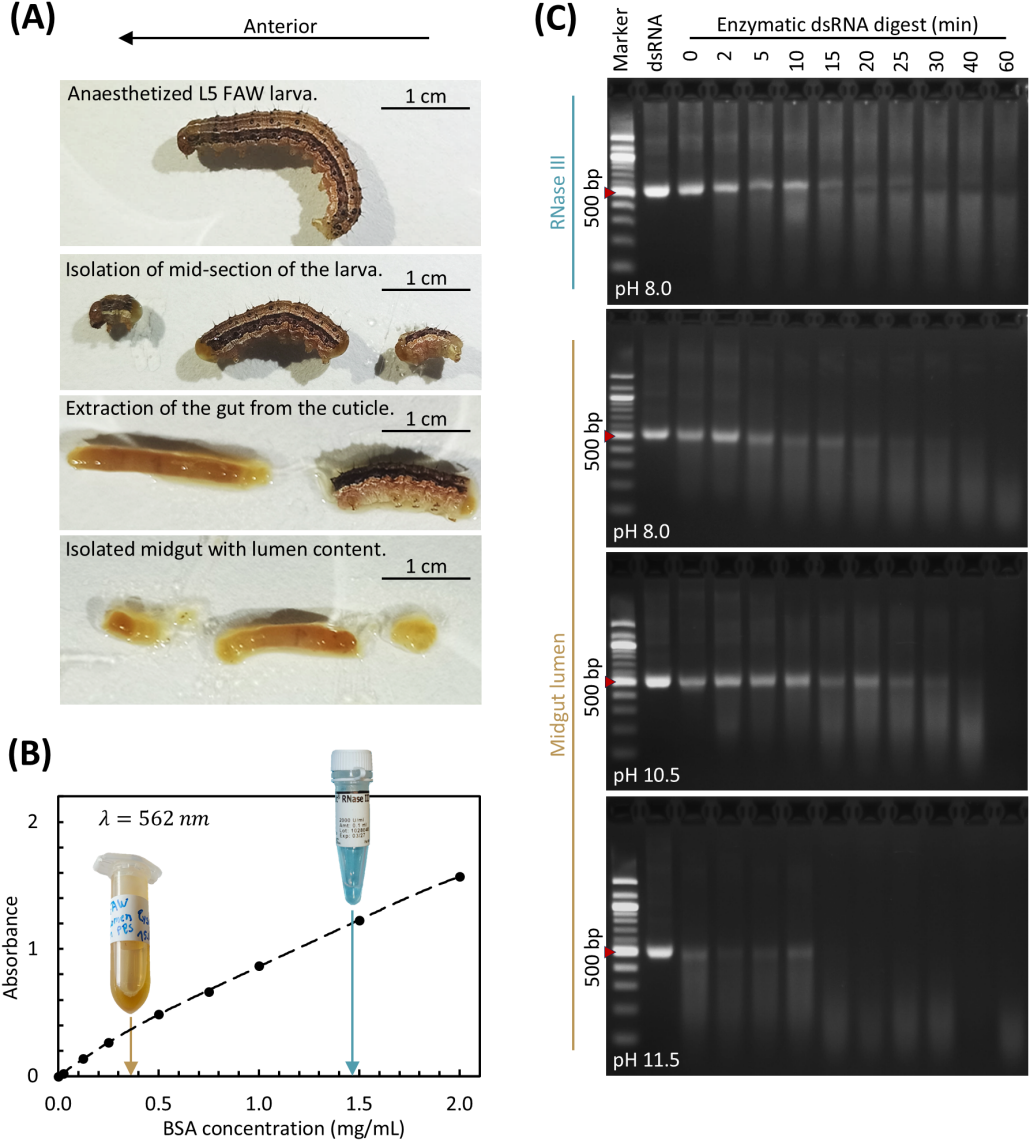
Alkaline pH enhances FAW midgut RNase activity, leading to accelerated dsRNA degradation compared to commercial RNase III. **(A)** Sequential images illustrating midgut dissection of a FAW L5 larva. (1) Anesthetized larvae were positioned in a Petri dish. (2) The head and posterior segments were removed to expose the midsection. (3) The cuticle was peeled back to reveal the midgut. (4) The foregut and hindgut were excised, isolating the midgut with its lumen contents. **(B)** Protein standard curve generated using the Pierce BCA Protein Assay Kit for protein quantification (BSA in PBS). Absorbance at 562 nm was plotted against BSA concentration (mg/mL), and a fourth-degree polynomial fit was applied as recommended by the manufacturer. Arrows indicate representative measurements of a 1:5 dilution of midgut lumen extract (orange arrow, 0.37 mg/mL; final protein concentration = 1.82 ± 0.04 mg/mL), and a commercial RNase III (blue arrow, 1.48 mg/mL; final protein concentration = 7.37 ± 0.28 mg/mL). **(C)** Time-resolved degradation of naked 500-bp dsRNA by FAW midgut lumen extract under different alkaline conditions. The dsRNA (4 µg) was incubated with 10 µg protein equivalent of midgut lumen extract or commercial RNase III at 32 °C, and reactions were stopped at defined time points (0–60 min) with SDS. Untreated dsRNA in water served as a control. Samples were analyzed by 2% agarose gel electrophoresis. Panels show identical time-course layouts for (1) RNase III at pH 8.0, (2) lumen extract at pH 8.0, (3) lumen extract at pH 10.5, and (4) lumen extract at pH 11.5. A 100-bp DNA ladder, with the 500-bp band indicated (red arrow), served as a size reference.

To evaluate RNase activity in FAW larvae, midgut lumen extracts were prepared from L5 specimens (**Figure 1A**). Filtered supernatants from homogenized and centrifuged lumen content were used as enzyme sources in subsequent dsRNA degradation assays. Extracts from the artificial diet were processed in parallel as controls to determine whether undigested material contributed to detectable enzymatic activity.

Protein concentrations in the lumen extract, artificial diet and commercial RNase III were quantified using a BCA protein assay, allowing the normalization of enzymatic activity to total protein content (**Figure 1B**). Standard curves for BSA in water and in PBS were fitted with fourth-degree polynomials (R² = 1.000 in both cases) to ensure accurate quantification. Under these conditions, the protein concentrations were 1.82 ± 0.04 mg/mL in the midgut lumen extract, 5.6 ± 0.3 mg/mL in the artificial diet extract, and 7.4 ± 0.3 mg/mL for RNase III.

Time-resolved dsRNA degradation assays at 32 °C revealed distinct activity profiles (**Figure 1C**). Reactions were standardized to 10 µg protein per sample. The commercial RNase III control at pH 8.0 produced rapid and complete degradation of 500-bp dsRNA, with a degradation smear appearing within 2 min and the intact dsRNA band disappearing after 15–20 min. A faint higher-molecular-weight band occasionally visible above the 500-bp dsRNA probably represented residual template DNA from dsRNA synthesis rather than degradation products because its intensity remained unchanged across treatments.

The FAW midgut lumen extract displayed robust activity at pH 8.0. The 500-bp dsRNA band graduallysmeared and diminished, disappearing completely after ~40 min, suggesting progressive fragmentation into smaller RNA fragments. To assess the impact of the naturally alkaline FAW gut environment, identical reactions were set up at pH 10.5 and 11.5. At pH 10.5, dsRNA degradation accelerated after 30 min, whereas fragmentation was pronounced from the earliest time points at pH 11.5, with near complete degradation observed after 40 min. The faint residual smear at 60 min may indicate the persistence of partially stabilized low-molecular-weight RNA species. Minor degradation was observed even at the start of the reaction across all pH conditions, probably reflecting spontaneous alkaline hydrolysis. No degradation was observed in the reactions containing artificial diet extract, confirming the absence of RNase activity in undigested diet material (**Supplementary Figure 2**).

### 3.2 Particle characteristics and protective properties

**Figure 2:**
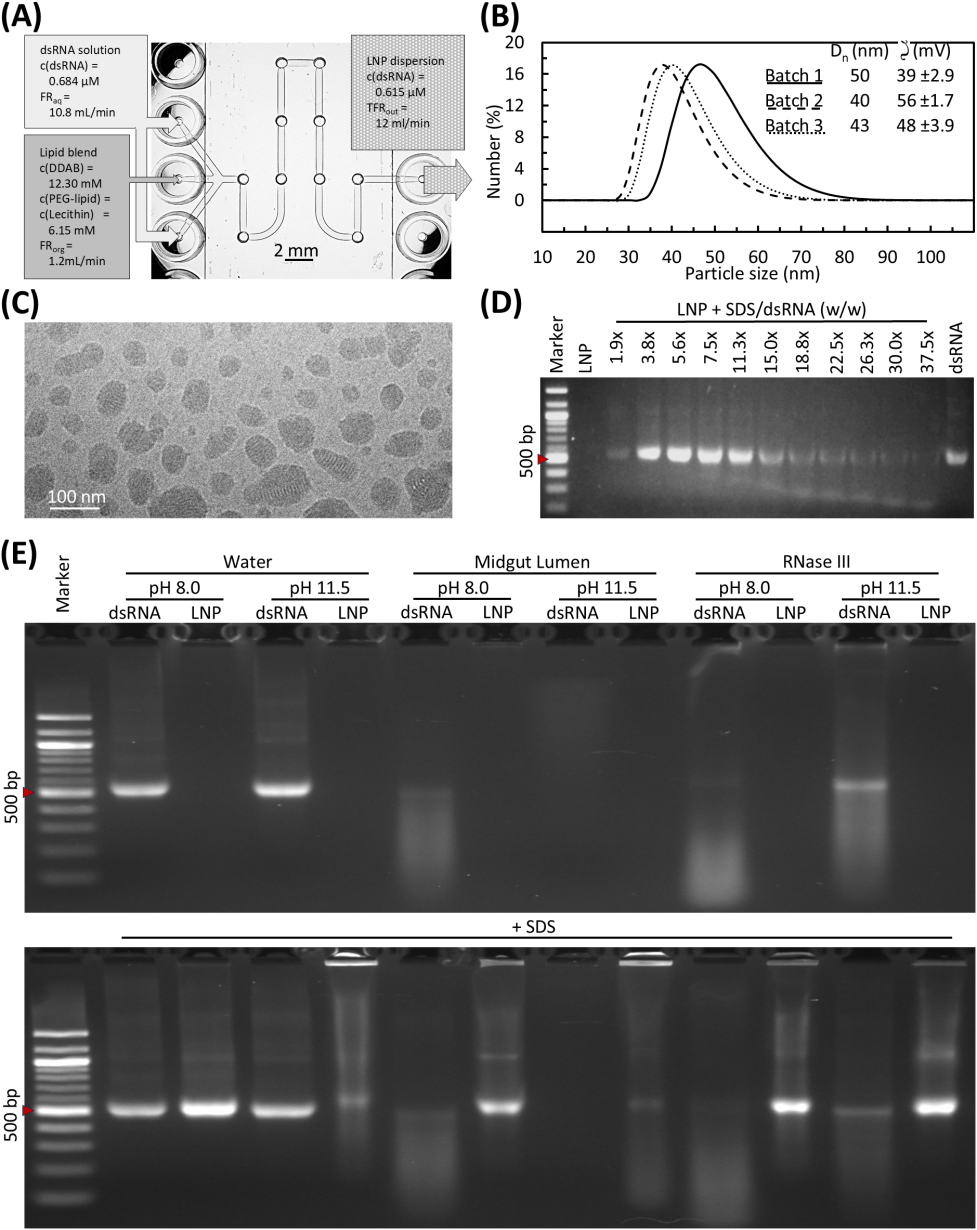
LNPs protect dsRNA from FAW midgut RNase activity, even under alkaline conditions. **(A)** Schematic of the serpentine microfluidic mixer used for LNP formulation. The aqueous dsRNA phase (0.222 g/L, 0.684 µM) entered through two outer inlets and the ethanolic lipid phase through the central inlet. Mixing in a 10-turn serpentine channel promoted chaotic advection and yielded LNP dispersions (final dsRNA = 0.2 g/L, L/P = 4; total flow = 12 mL/min). Scale bar = 2 mm. **(B)** Particle size distribution and surface charge characterization of three LNP batches measured by MADLS. Mean particle diameters (D_n_) ranged from 40 to 50 nm (batch 1, 50 nm; batch 2, 40 nm; batch 3, 43 nm), with strongly positive zeta (ζ) potentials (39 ± 2.9, 56 ± 1.7, 48 ± 3.9 mV). **(C)** Cryo-TEM images of representative dsRNA–LNP formulations showing predominantly spherical particles within the dominant ~35–60 nm size range together with populations of smaller and larger particles, consistent with MADLS size distributions. Scale bar = 100 nm. **(D)** SDS-induced release of 500-bp dsRNA from LNPs visualized by 2% agarose gel electrophoresis. The untreated dsRNA–LNP control (LNP) shows no detectable fluorescence, indicating intact complexation. SDS was added at defined mass ratios relative to a constant dsRNA concentration (e.g., 3.8×, 5.6×), with identical volumes across samples. Increasing SDS/dsRNA ratios led to progressive nanoparticle decomplexation and the appearance of the 500-bp band, whereas band intensity declined at the highest SDS ratios due to stain wash-out. The appearance of less-intense bands at high SDS concentrations reflects diminished SYBR Gold staining efficiency under strongly anionic conditions. The integrity of dsRNA in the presence of SDS was confirmed using naked dsRNA controls as shown in panel (E). The dsRNA control appears at the expected position. A 100-bp DNA ladder is included with the 500-bp marker indicated (red arrow). **(E)** pH-dependent digestion of naked (dsRNA) and LNP-formulated (LNP) 500-bp dsRNA by FAW midgut lumen extract or RNase III (10 µg protein, 32 °C, 60 min) at pH 8 and pH 11.5. Water-only controls were included for each pH condition. The upper panel shows untreated reactions and the lower panel shows identical reactions stopped with SDS (3.8× w/w relative to dsRNA) to halt enzymatic activity and release dsRNA from LNPs. Samples were analyzed by 2% agarose gel electrophoresis with a 100-bp ladder (500-bp band indicated by red arrow). The FAW midgut extract retained stronger dsRNA-degrading activity than commercial RNase III under alkaline conditions. LNPs provided extended protection against enzymatic degradation even at highly alkaline pH.

To evaluate the suitability of LNPs for dsRNA delivery, their physicochemical properties and encapsulation capacity were analyzed before enzymatic assays. The dsRNA-loaded LNP formulations appeared visually uniform, presenting as slightly milky to opalescent suspensions without visible precipitates or aggregates. MADLS analysis confirmed the reproducibility of the microfluidic mixing method (**Figure 2A**), with the dominant particle population of each batch detected in the 40–50-nm range by number-based analysis (**Figure 2B**). Correspondence between number- and intensity-weighted distributions indicated low aggregation and overall formulation uniformity. The intensity-weighted size distributions were similar across all three batches, with main peaks of 50–60 nm. Only the first batch showed a minor secondary peak around 456 nm, corresponding to a small fraction of larger aggregates (**Supplementary Figure 1A**).

We observed a moderate increase in apparent particle diameter for LNPs prepared with Cy3-labeled dsRNA (91 nm by number, 105 nm by intensity; **Supplementary Figure 1B**), which probably reflects the covalent linkage of the Cy3 fluorophore to the phosphate backbone, thereby altering the surface charge and hydrodynamic properties of the dsRNA. The zeta potential of all unlabeled LNP batches revealed consistently high positive surface charges (+39 to +56 mV), indicating good colloidal stability and minimal aggregation during storage (**Figure 2B**).

Cryo-TEM imaging confirmed the structural integrity and nanoscale morphology of representative LNPs (**Figure 2C**). Particles were predominantly spherical to mildly ellipsoidal, with the majority ofdiameters falling within the ~40–60 nm range, consistent with the dominant number-based MADLS population. Smaller particles (~20–30 nm) and occasional larger particles (> 80 nm) were also observed, reflecting the expected polydispersity of lipid nanoparticle systems and corresponding to minor fractions present in the broader intensity-weighted size distributions. The diameters observed in vitrified samples closely matched the size range detected by MADLS, noting that cryogenic imaging reports physical particle dimensions in the absence of hydrodynamic effects. Many nanoparticles contained characteristic internal lamellar and electron-dense domains, consistent with tightly packed lipid–RNA complexes formed during microfluidic assembly. We also observed a small number of empty, spherical vesicles, corresponding to non-loaded lipid structures typically present in ionizable or cationic LNP formulations (**Supplementary Figure 3A**). In addition, rare larger aggregates appeared in some micrographs, aligning with the minorsecondary MADLS peak (~456 nm) detected in the first batch (**Supplementary Figure 3B**). Overall, the cryo-TEM images supported the colloidal and structural consistency of the LNPs indicated by MADLS and zeta potential measurements.

To evaluate the efficiency of encapsulation and the controlled release of dsRNA, we used SDS to induce LNP decomplexation. In the absence of SDS, LNP-formulated dsRNA produced no visible band, confirming efficient encapsulation. In the presence of SDS, a distinct 500-bp dsRNA band gradually emerged when the SDS/dsRNA weight ratio exceeded 1.9:1, corresponding to the progressive release of dsRNA as lipid–RNA interactions were disrupted (**Figure 2D**). Band intensity increased with moderate SDS concentrations, indicating efficient release, but diminished again at higher SDS concentrations. The reduced band intensity observed at higher SDS concentrations reflects impaired SYBR Gold staining under stronger anionic conditions rather than dsRNA degradation, as confirmed by intact naked dsRNA controls treated with SDS at both pH values (**Figure 2E**). Based on these results, an SDS/dsRNA weight ratio of 3.8:1 was chosen for subsequent decomplexation steps as the minimal concentration ensuring complete LNP disruption and dsRNA recovery while simultaneously inactivating enzymes.

To evaluate whether LNPs confer protective stability under conditions mimicking the highly alkaline lepidopteran midgut, dsRNA degradation assays were performed at pH 8.0 and 11.5 using FAW midgut lumen extracts and commercial RNase III. At pH 8.0, naked dsRNA was efficiently degraded by both enzyme sources. At pH 11.5, however, pronounced degradation was observed only with the midgut lumen extract, consistent with its elevated activity under physiological FAW midgut conditions and the complete digestion detected at the 40-min and 60-min time points (pH 10.5 and 11.5) in **Figure 1C**. In contrast, LNP-formulated dsRNA remained undetectable during incubation at both pH values and became visible only following SDS-induced decomplexation, demonstrating effective encapsulation and protection from enzymatic and alkaline degradation. Although SDS addition and high pH induced pronounced “wash-out” effects characteristic of SDS-rich conditions, the presence of intact 500-bp bands following decomplexation confirmed that the LNPs maintained the integrity of the dsRNA throughout incubation (**Figure 2E**).

### 3.3 FAW uptake of Cy3-labeled dsRNA is enhanced by LNP formulation

To assess whether LNP formulation enhances the cellular uptake and systemic distribution of dsRNA in FAW larvae, we fed groups of larvae on diets supplemented with Cy3-labeled 500-bp dsRNA (naked or encapsulated within LNPs) and traced the fluorescence from the midgut lumen across the peritrophic matrix and epithelial barrier into the hemocoel. Larvae were starved for 4 h before feeding on lyophilized artificial diet pellets containing 2.5 µL (per 30 µL of pellet per larva) of naked dsRNA-Cy3, LNP-formulated dsRNA-Cy3 or water as a control.

**Figure 3:**
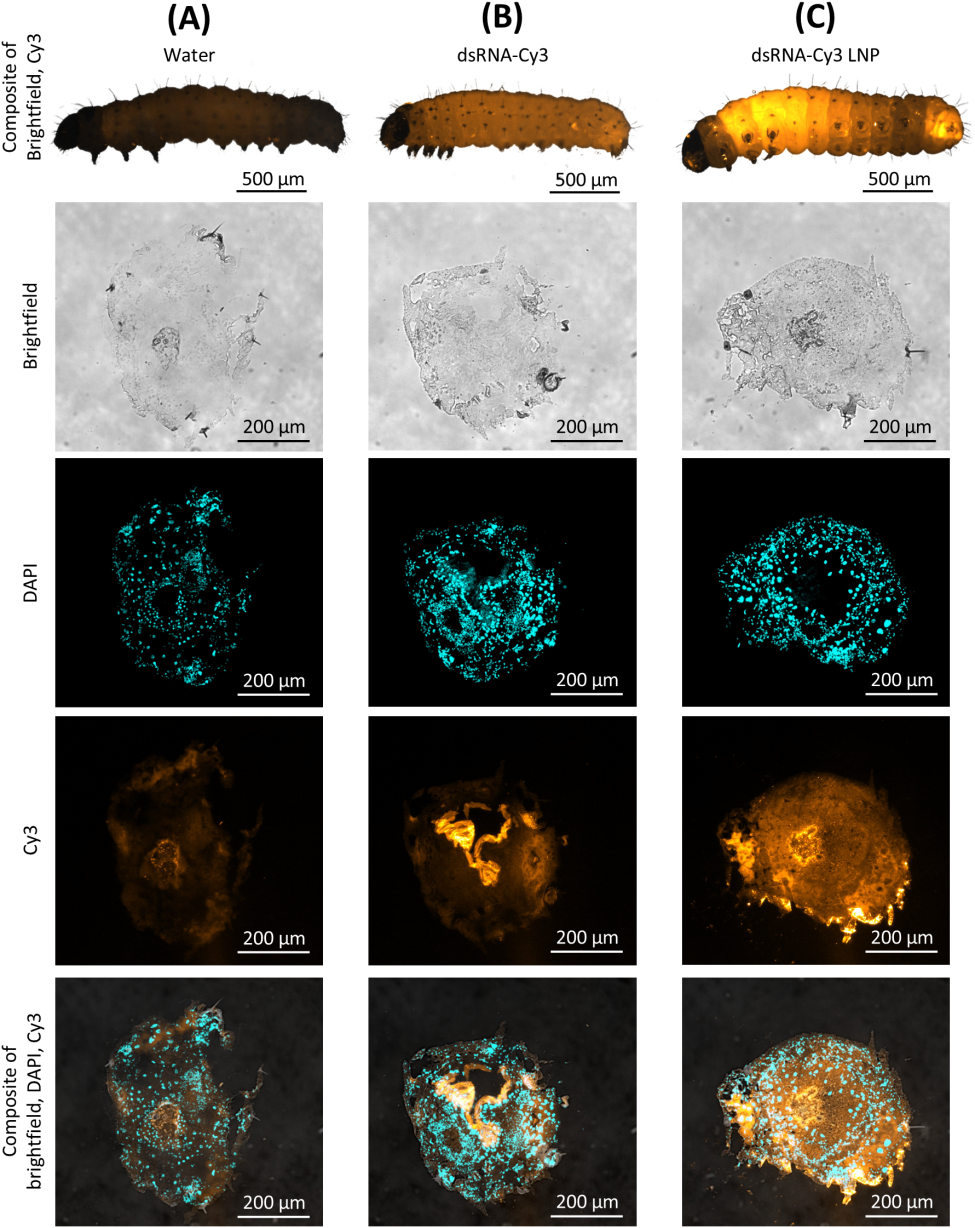
Tissue distribution of Cy3-labeled dsRNA fed to L1 FAW larvae. Fluorescence microscopy revealed treatment-dependent differences in dsRNA localization. **(A)** Water control showing minimal Cy3 background signal (autofluorescence). **(B)** Naked dsRNA-Cy3 signal restricted to the gut lumen and peritrophic membrane, indicating limited penetration into surrounding tissues. **(C)** LNP-formulated dsRNA-Cy3 signal extending beyond the midgut epithelium, consistent with enhanced tissue uptake. For each treatment, panels depict a whole-larva view (merged bright-field/Cy3; scale bar = 500 µm) and transverse midgut sections imaged in bright-field, DAPI, Cy3, and merged channels (scale bars = 200 µm). Microscope settings were identical across samples, and identical post-processing adjustments were applied uniformly to all images for visualization.

Fluorescence microscopy revealed differences in the tissue distribution of fluorescence between treatment groups in cross-sections of L1 larvae (**Figures 3A–C**). Only weak background autofluorescence was observed in the water control, confirming minimal interference with Cy3 detection (**Figure 3A**). Whole-larva imaging confirmed that larvae from all dsRNA-fed treatment groups exhibited Cy3 fluorescence within the gut lumen, indicating successful ingestion under the standardized feeding conditions we used. The naked dsRNA-Cy3 signal was detected primarily in the gut lumen and especially along the peritrophic matrix, with only a faint signal beyond the gut epithelium, indicating limited trans-epithelial transport (**Figure 3B**). In contrast, LNP-formulated dsRNA-Cy3 generated a stronger and more extensive signal, spreading beyond the midgut epithelium into surrounding tissues (**Figure 3C**). This broader pattern suggests that LNP encapsulation facilitates the movement of dsRNA and/or its fragments across the intestinal barrier and into the hemocoel. Representative images are shown in **Figure 3**, and complete image series for additional larvae are provided in **Supplementary Figures 4**-**7**. These results demonstrate that LNP formulation enhances dsRNA uptake and systemic spreading within FAW larvae, confirming the potential of LNPs for improved RNAi delivery.

**Figure 4:**
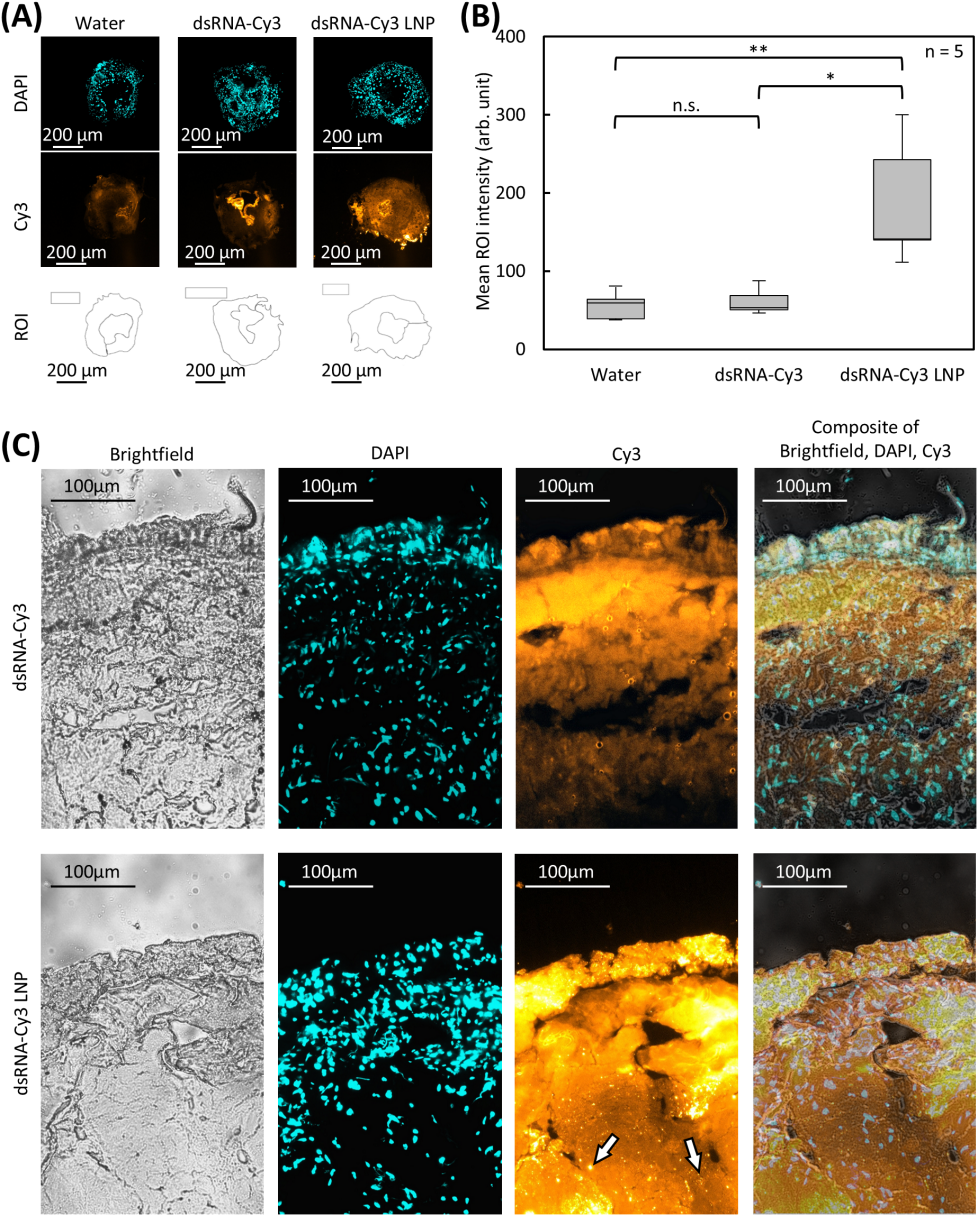
Quantitative and tissue−level analysis of dsRNA−Cy3 uptake in FAW larvae. **(A)** Fluorescence quantification workflow in L1 larvae fed with water (control), naked dsRNA−Cy3, or LNP−formulated dsRNA−Cy3. Representative transverse cryosections show DAPI−stained nuclei (tissue structure and hemocoel identification), Cy3 fluorescence (labeled dsRNA), and manually defined regions of interest (ROIs) excluding the midgut lumen and peritrophic membrane. The signal was quantified on raw images with identical exposure settings and display brightness was uniformly adjusted for clarity. Scale bars = 200 µm. **(B)** Quantification of Cy3 fluorescence intensity in the hemocoel of L1 larvae (n = 5). Boxplots show background−corrected mean Cy3 intensities (arbitrary units) after feeding with water, naked dsRNA−Cy3, or LNP−dsRNA−Cy3. ROIs were defined on the DAPI channel for hemocoel identification, and background was measured outside the larval body. Statistical significance (paired t−test, α = 0.05; *p < 0.05, **p < 0.01): water vs LNP (p = 0.0064), naked vs LNP (p = 0.0121), water vs naked (n.s.). **(C)** Uptake of dsRNA−Cy3 and LNP−formulated dsRNA−Cy3 by dissected L4 midgut epithelium. Midguts were incubated for 1 h at 32 °C with 4 µL of naked or LNP−formulated dsRNA−Cy3 (0.1 µg/µL). Transverse cryosections (16 µm) stained with DAPI show bright-field, DAPI, Cy3, and merged channels. Naked dsRNA−Cy3 fluorescence was largely confined to subepithelial regions (~100–150 µm deep) whereas LNP−formulated dsRNA−Cy3 showed deeper penetration and formed fluorescent agglomerates (1–10 µm) and localized uptake hotspots indicated by white arrows. Scale bars = 100 µm.

We quantified the dsRNA distribution in each treatment group by measuring the fluorescence intensity in defined ROIs within transverse midgut sections of the larvae. ROIs were manually delineated on DAPI-stained images, excluding the midgut lumen and peritrophic membrane, which lack nuclei and therefore do not represent tissue-associated signals (**Figure 4A**).For each individual larva (n = 5 per treatment), the same ROIs were then applied to the Cy3 channel to determine mean fluorescence intensity (range 0–4095). The background signal, measured from ROIs placed outside the larvae, was subtracted from all values. Statistical analysis revealed no significant difference between water and naked dsRNA-Cy3 treatments (p = 0.3592). In contrast, the LNP-formulated dsRNA-Cy3 produced significantly higher fluorescence intensity in the hemocoel compared to both water (p = 0.0064) and naked dsRNA (p = 0.0121), indicating enhanced uptake and systemic transport of dsRNA or its labeled degradation products facilitated by LNP encapsulation (**Figure 4B**).

To investigate midgut penetration in more detail, dissected midguts from L4 larvae were opened sagittally and incubated lumen side uppermost for 1 h at 32 °C in a wet chamber with either naked or LNP-formulated dsRNA-Cy3 (**Figure 4C**). Bright-field panels confirmed comparable tissue integrity and cryosection quality across treatments. DAPI-stained nuclei provided a spatial reference for epithelial and subepithelial regions. Fluorescence microscopy revealed that naked dsRNA-Cy3 localized primarily just below the epithelial surface, with signal intensity rapidly declining beyond a depth of ~100–150 µm. In contrast, LNP-formulated dsRNA-Cy3 formed discrete fluorescent foci ranging from ~1 µm to 10 µm in diameter, and formed distinct high-intensity regions within deeper tissue layers. These findings indicate that LNP formulation enhances the delivery and trans-epithelial penetration of dsRNA within FAW midgut tissues.

## 4 Discussion

A central paradox in the use of RNAi against agricultural pest insects is that nucleases play both antagonistic and beneficial roles in the pathway. On one hand, extracellular RNases in the insect midgut rapidly degrade orally delivered dsRNA into short fragments that are too small to trigger systemic RNAi efficiently, thereby contributing to the poor responsiveness observed in many lepidopterans. These truncated products often fall below the threshold length required for effective uptake, intracellular processing, and amplification. Multiple studies have demonstrated a strictly length-dependent efficacy: long dsRNAs (≥ 200–400 bp) consistently induce far stronger knockdown effects than shorter fragments, whereas dsRNAs of 60 bp or shorter are largely ineffective under physiological exposure conditions (49, 50). On the other hand, within the RNAi pathway itself, RNA processing enzymes such as Dicer-2 are indispensable because they generate the functional 21–23-nt siRNAs that facilitate target mRNA cleavage. Inefficient RNAi in many lepidopterans thus reflects the interplay between these opposing forces: strong midgut RNase activity breaks down dsRNA substrates before Dicer-2 can act, but Dicer-2 expression in gut tissues may be intrinsically low (51), limiting the conversion of ingested dsRNA into functional siRNAs. Together, these constraints underscore the importance of protecting long dsRNA molecules during midgut transit to preserve their integrity and enable productive engagement with the intracellular RNAi machinery.

The degradation assays revealed that FAW midgut RNase activity is maintained even at mildly alkaline pH, with degradation accelerating at pH 11.5, which represents the native midgut environment in FAW larvae (52, 53). RNases are therefore the primary extracellular barriers to RNAi, a conclusion supported by earlier reports showing several midgut-expressed nucleases with basophilic activity profiles and optimal catalysis at pH 9–10 (54, 55). The extreme alkalinity of the lepidopteran midgut, often exceeding pH 11, enhances the chemical hydrolysis of dsRNA as well as supporting native enzymes. Such conditions explain the rapid disappearance of the intact 500-bp dsRNA band in our *ex vivo* assay and contribute to the overall RNAi recalcitrance frequently reported in lepidopterans (56).

The encapsulation of dsRNA mitigates its sensitivity to alkaline hydrolysis and gut enzymes (57, 58). The formulated LNPs conferred substantial protection against degradation by both FAW enzyme extracts and commercial RNase III. Following SDS-mediated decomplexation, intact 500-bp dsRNA bands were recovered, confirming that the lipid matrix provided steric and electrostatic shielding from nuclease attack. Occasional “wash-out” effects in gels probably reflected SDS-related artifacts (hydroxide and surfactant anions interacting with the cationic stain) rather than degradation. These results suggest that a positively charged lipid shell (primarily imparted by DDAB) serves a dual function by stabilizing particles via electrostatic repulsion and also shielding anionic dsRNA from enzymes (59, 60).

The physicochemical analysis of the three LNP batches demonstrated excellent reproducibility and colloidal stability. The mean particle size of 40–60 nm and strong positive zeta potential (+39 to +56 mV) conform to design criteria known to favor effective interaction with negatively charged cell membranes in the gut (61, 62). Nanoparticles with a diameter < 100 nm can traverse the peritrophic matrix and are more efficiently internalized by endocytosis than larger particles (63, 64), which tend to be trapped in the gut lumen. The microfluidic preparation method was able to generate uniform LNPs with these optimized properties. A moderate size increase was detected for Cy3-labeled dsRNA formulations, probably reflecting the altered charge distribution during LNP self-assembly, emphasizing the need to account for fluorophore-induced physicochemical changes in uptake studies.

Microscopy provided direct visual evidence for improved dsRNA delivery mediated by LNPs. Larvae fed with LNP-formulated dsRNA-Cy3 displayed markedly stronger and more widespread fluorescence than those fed with naked dsRNA. Signals extended beyond the peritrophic membrane and the midgut epithelium into the hemocoel, indicating successful trans-epithelial passage. Quantitative image analysis confirmed significantly higher fluorescence intensity in the hemocoel ROIs of LNP-fed larvae, demonstrating that LNP delivery enhances the systemic transport of dsRNA or its labeled degradation products. These findings were supported by *ex vivo* midgut incubation experiments, which showed the deeper tissue penetration of LNP-formulated dsRNA compared to naked dsRNA, including the presence of discrete intracellular fluorescent agglomerates and localized hot spots up to 10 µm in diameter. The punctate intracellular distribution pattern suggests vesicular uptake and is consistent with the endosomal compartmentalization of LNP-associated dsRNA (65, 66). However, definitive confirmation of endosomal trafficking or endosomal escape will require co-localization with organelle-specific markers or live-cell tracking approaches.

The efficiency of this delivery strategy probably reflects a combination of small particle size and high cationic surface charge, which facilitate adhesion to anionic epithelial membranes and promote endocytic uptake (58, 67, 68). The protective lipid shell prevents rapid nuclease degradation prior to uptake, thereby addressing two major physiological barriers simultaneously. Although the cationic nature of DDAB-rich formulations can raise concerns regarding cytotoxicity or immune activation, particularly under repeated exposure scenarios, systematic assessment of LNP-only effects on larval survival and development will be required in future feeding assays optimized for solvent-free or evaporated formulations. Notably, ethanol present in formulation buffers can influence larval feeding behavior in enclosed assay systems, emphasizing the need for ethanol-free or lyophilized formulations prior to quantitative efficacy and toxicity studies.

### 4.1 Conclusion and outlook

We have shown that low-cost cationic LNPs with an appropriately sized dsRNA cargo enhance the efficiency of RNAi in FAW larvae by protecting the dsRNA from enzymatic and chemical degradation and promoting its epithelial translocation after ingestion. These results advance our understanding of biological and physicochemical parameters that limit the potential of oral RNAi in lepidopterans and mark progress toward the rational design of nanocarriers for RNA-based pest control despite the challenging conditions of the insect digestive tract. We did not evaluate gene knockdown efficacy because the primary aim was to establish a delivery platform capable of overcoming physiological barriers. Functional RNAi assays will be carried out in future studies.

The refinement and exploration of complementary nanocarrier technologies is essential. Future research should systematically investigate formulation parameters that confer dsRNA stability and modulate release kinetics, particularly under field-relevant conditions. Promising comparative approaches include chitosan/dsRNA complexes with additive insecticidal effects (59, 60), metal-organic frameworks and polydopamine-coated nanoparticles (69), as well as pH-responsive and poly-l-arginine-functionalized nanocarriers (63, 70).

Controlled feeding assays using fluorescence-labeled or radiolabeled dsRNA could be used to track nanoparticle transit, uptake kinetics, and systemic distribution with high precision, providing quantitative insight into dsRNA delivery efficiency (71). However, establishing that improved delivery translates into robust gene knockdown and measurable fitness costs *in vivo* will be necessary to validate LNP-enabled RNAi in lepidopteran pests. Identifying the thermodynamic and electrostatic parameters governing dsRNA–lipid complexation will also facilitate rational nanocarrier design. In parallel, combining optimized LNP formulations with the selection of highly responsive gene targets guided by recent RNAi screening in FAW and related lepidopterans (51, 72, 73) will substantially improve gene silencing outcomes. Ultimately, only the convergence of nanocarrier engineering, molecular target selection, and rigorous biological efficacy assays will determine whether oral RNAi can be translated into reliable, species-restricted biopesticides capable of overcoming the intrinsic physiological barriers of lepidopteran pests (74–76).

## Data Availability

The original contributions presented in the study are included in the article/**Supplementary Material**. Further inquiries can be directed to the corresponding author.
