# Supplementary figures and images for: Enhancing the delivery and stability of lipid nanoparticle–dsRNA formulations in the RNAi-recalcitrant fall armyworm (Spodoptera frugiperda)
Source: Front Insect Sci. 2026 Mar 26;6:1770055. doi: 10.3389/finsc.2026.1770055 (PMC13062331; doi:10.3389/finsc.2026.1770055)

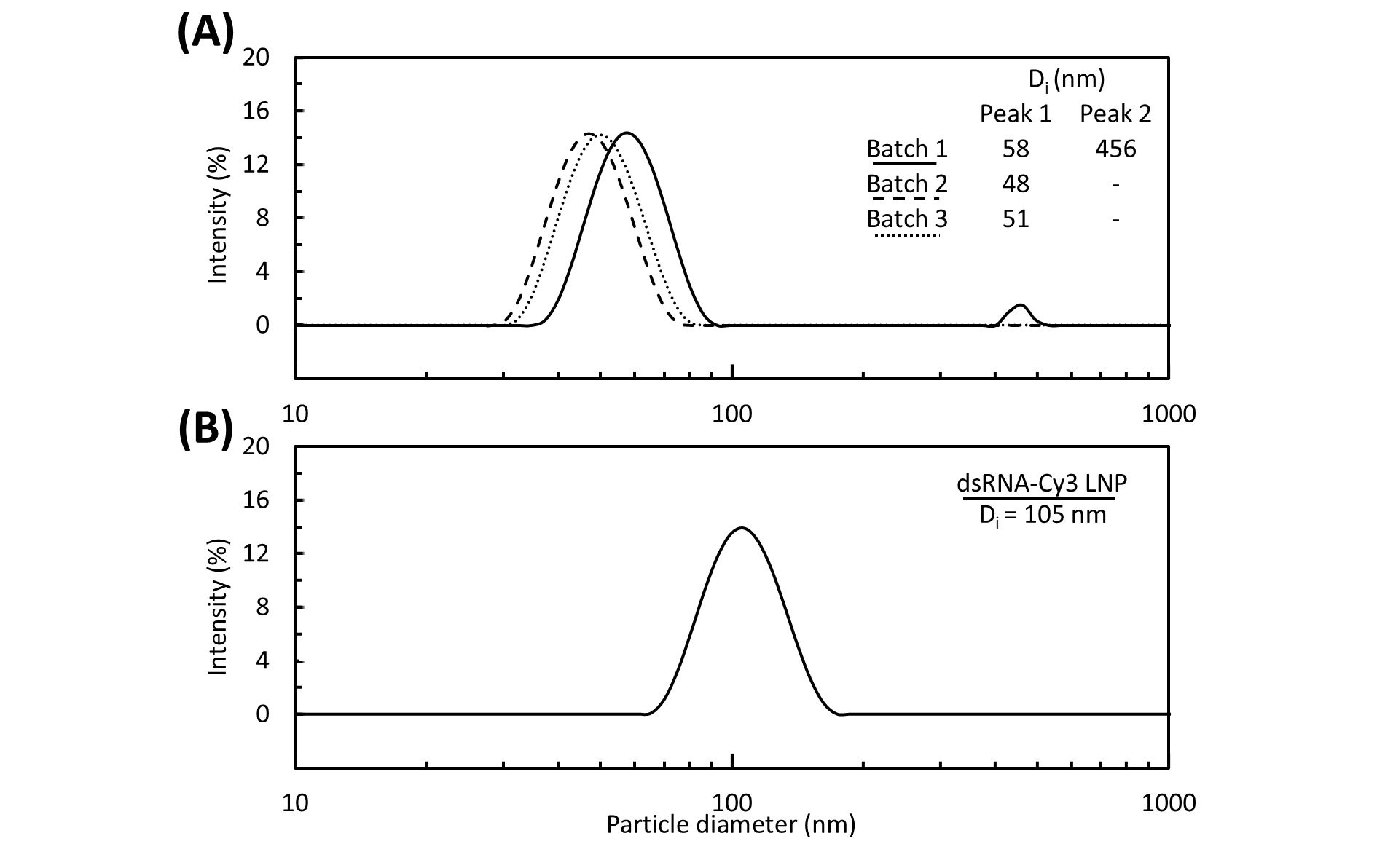

Supplement: Supplementary file 1 [file Image1.tif]

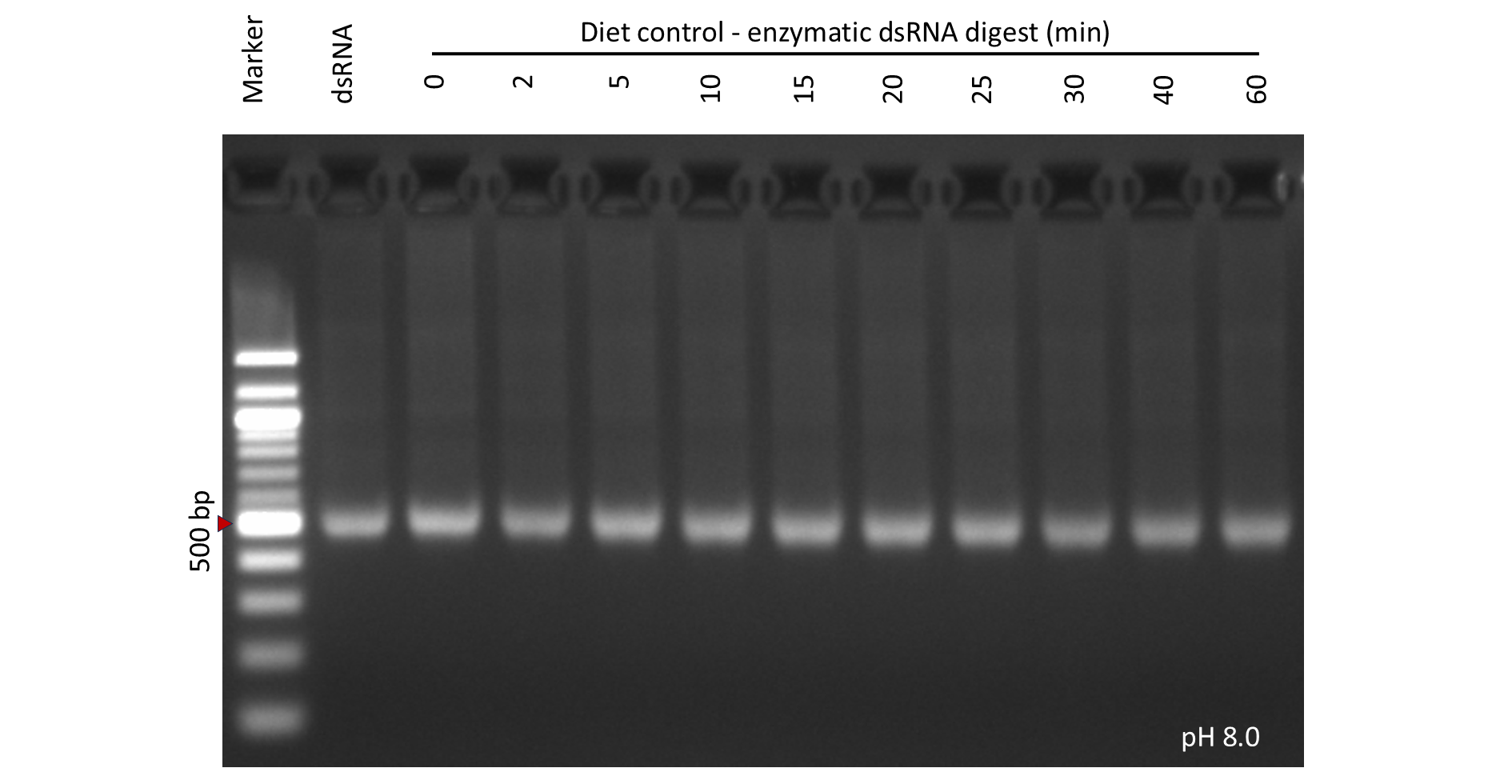

Supplement: Supplementary file 2 [file Image2.tif]

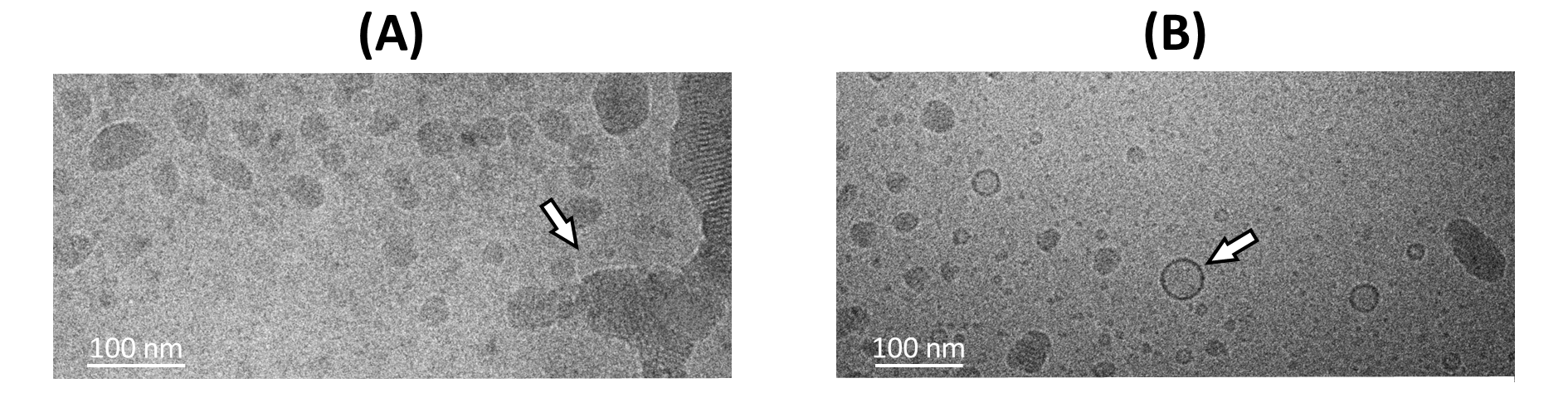

Supplement: Supplementary file 3 [file Image3.tif]

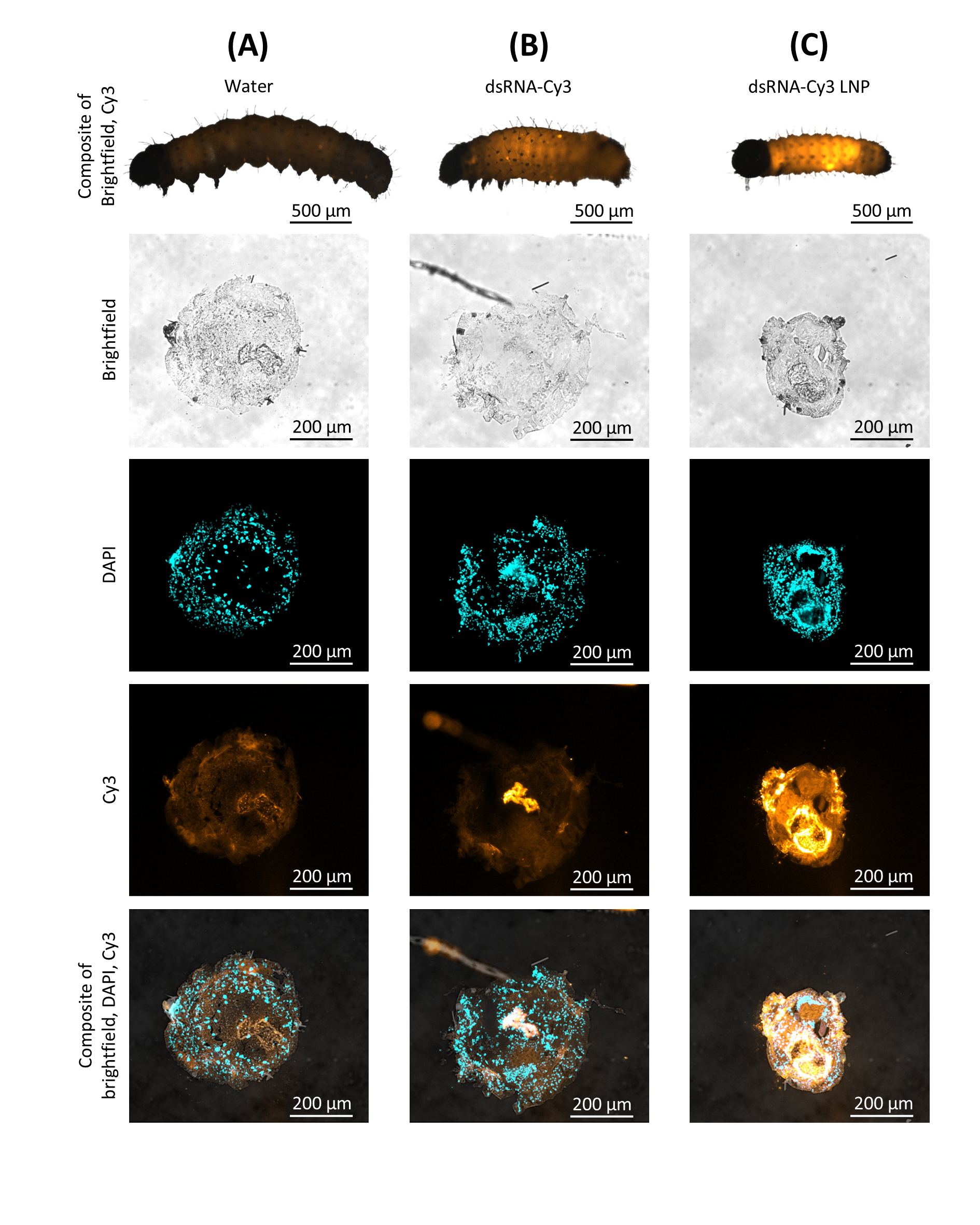

Supplement: Supplementary file 4 [file Image4.tif]

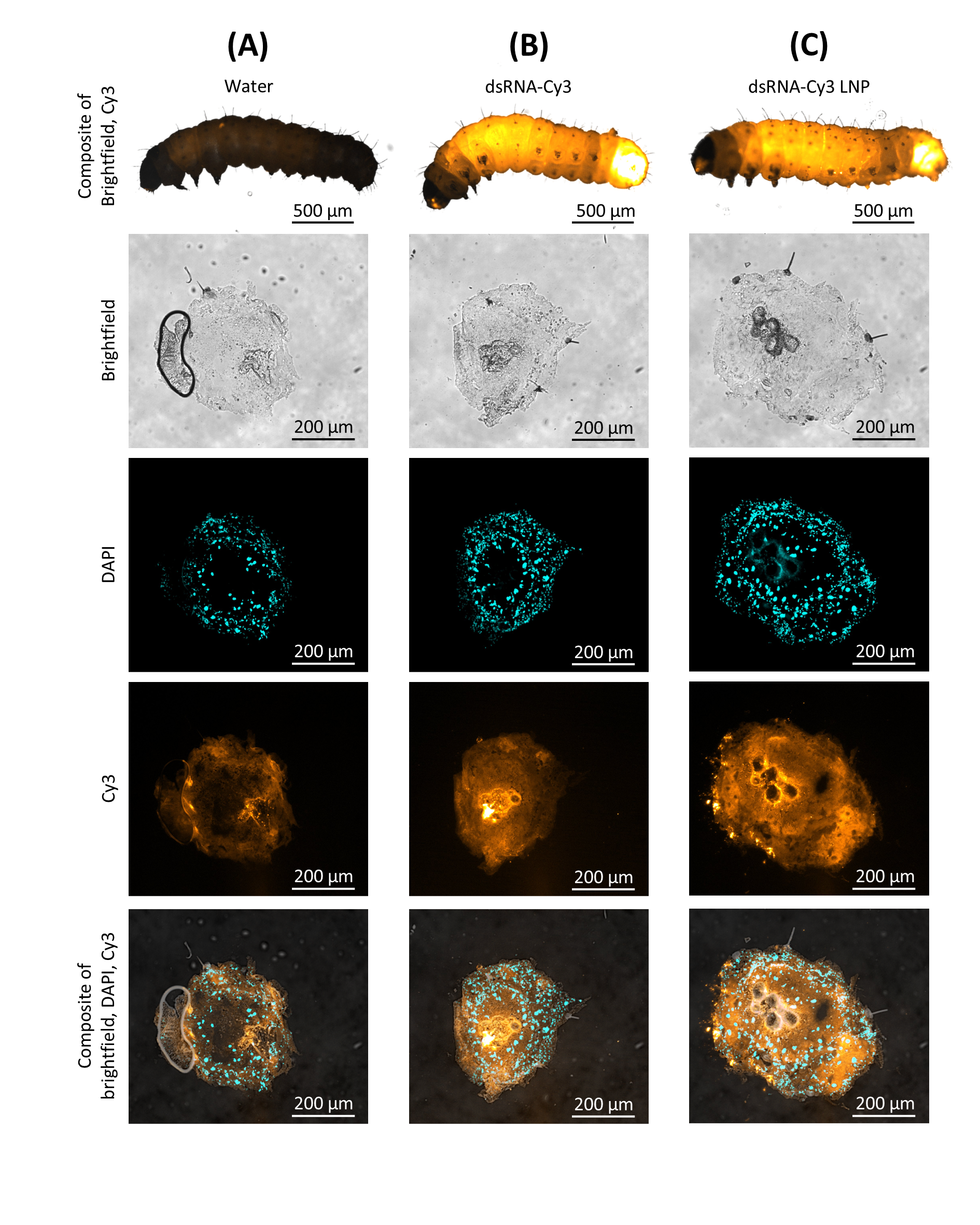

Supplement: Supplementary file 5 [file Image5.tif]

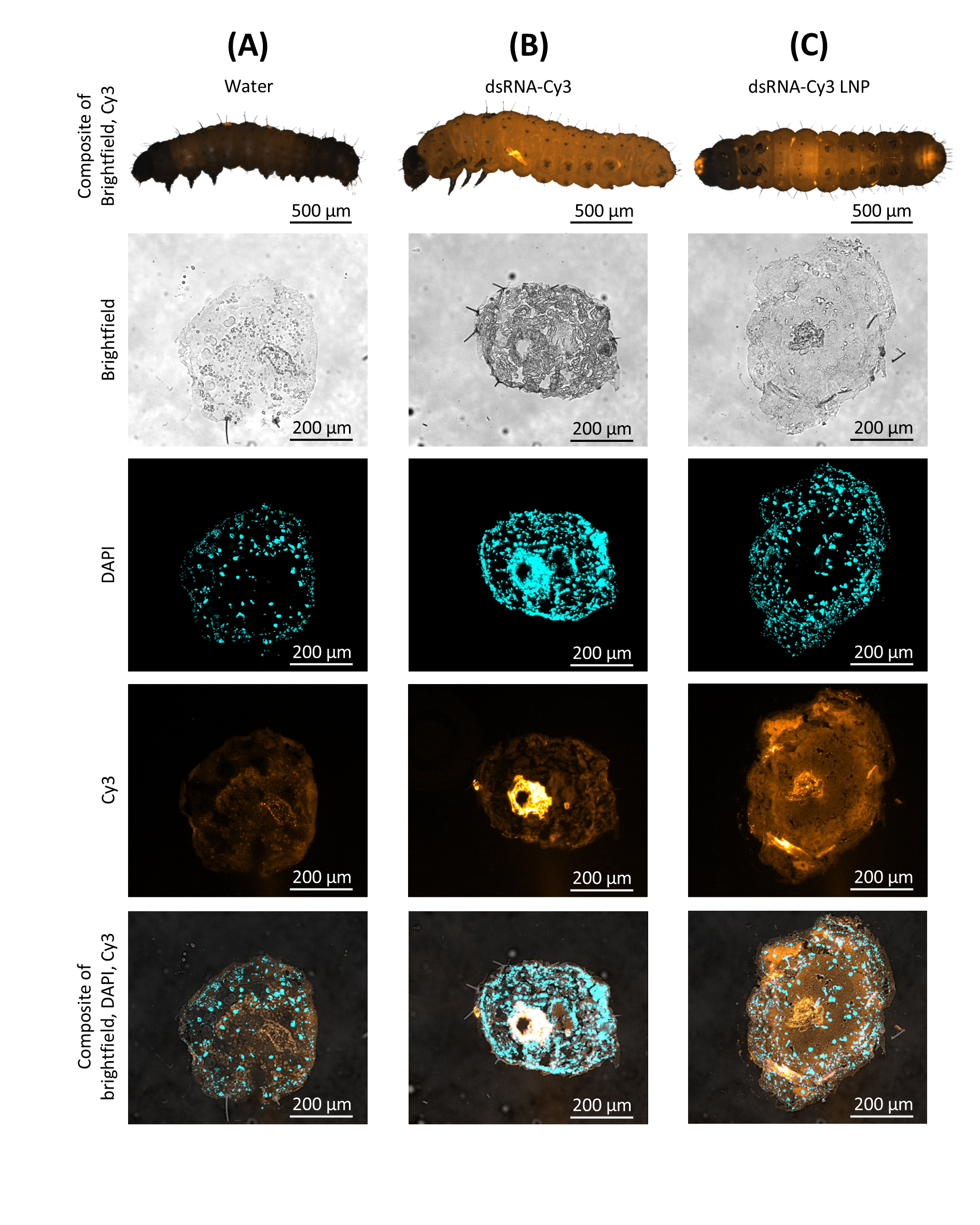

Supplement: Supplementary file 6 [file Image6.tif]

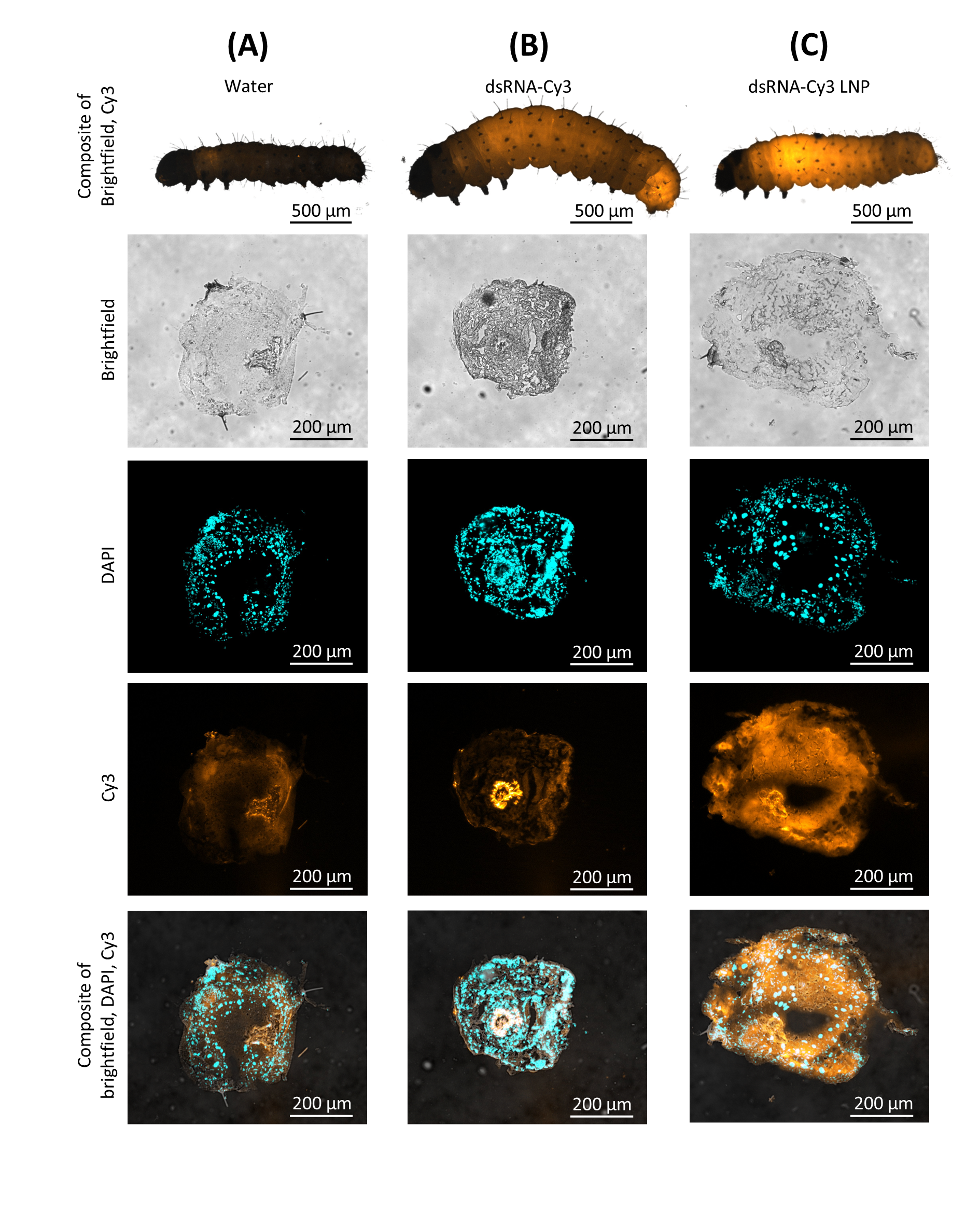

Supplement: Supplementary file 7 [file Image7.tif]
